# Impact of scheduled partial exchange transfusions on outcomes in pregnant patients with severe sickle cell disease: a retrospective study

**DOI:** 10.1016/j.htct.2024.07.001

**Published:** 2024-09-07

**Authors:** Anil Ananthaneni, Sarah Jones, Mohamed Ghoweba, Vishwa Grant, Kenna Leethy, Taras Benzar, Samip Master, Richard Mansour, Poornima Ramadas

**Affiliations:** a Louisiana State University Health Sciences Center Shreveport, Shreveport, LA, USA; b Vascular and Thoracic Institute, Cleveland Clinic Foundation, Cleveland, OH, USA; c Phoenix Children's Hospital, Phoenix, AZ, USA

**Keywords:** Pregnancy, Sickle cell disease, SCD, Red cell exchange, Transfusion, Outcome

## Abstract

**Introduction:**

The incidence of feto-maternal complications is high in women with sickle cell disease. The paucity of high-quality evidence has led to conditional recommendations for transfusional support in pregnant patients. This study aimed to assess if scheduled partial red cell exchanges impact pregnancy outcomes in sickle cell disease patients.

**Methods:**

Forty-seven pregnancies were divided into two groups based on whether patients received scheduled partial red cell exchanges. Collected data included demographics, laboratory values, number of hospital visits, and prenatal/perinatal/postnatal outcomes. Data were analyzed using descriptive statistics, t-test, Chi-square and Fisher's exact tests, and binary regression.

**Results:**

The mean age was 25.09 ± 4.39 years. Of 47 patients, 14 (29.8%) received scheduled red cell exchanges with 78.6% compliance with no evidence of alloimmunization. This procedure during pregnancy was associated with fewer admissions for pain crises (p=0.032), higher gestational age at delivery (p=0.007), and a lower incidence of neonatal intensive care admissions (p=0.011; odds ratio: 0.071; 95% confidence interval: 0.008-0.632). Logistic regression did not show any significant associations.

**Conclusions:**

Sickle cell disease patients with complications in previous pregnancies, including high hospitalization/admission rates and preterm deliveries, could benefit from scheduled partial red cell exchanges or simple transfusions. Further research is needed to guide clinical practice pertaining to transfusional support in pregnant patients with sickle cell disease.

## Introduction

Sickle cell disease (SCD) is the most common inherited blood disorder in the United States, with an estimated prevalence of around 100,000 individuals. It affects about 1 in 500 African Americans, and 1 in 1000-1400 Hispanic Americans.^1^^,^^2^ Common acute complications include vaso-occlusive crises, which encompass painful crises, acute chest syndrome, and strokes. Chronic complications include anemia, chronic kidney disease, heart failure, pulmonary hypertension, retinopathy, and neurocognitive dysfunction, among others.^3^ Pregnancy in SCD patients poses an additional risk of increased frequency of vaso-occlusive events, anemia, maternal mortality, preeclampsia, placental abruption, stillbirth, preterm delivery, and small for gestational age infants.^4^, ^5^, ^6^, ^7^

Red blood cell transfusions play a significant role in the acute and chronic management of complications associated with SCD.^8^ In pregnant patients with SCD, transfusional therapy is an actively debated topic due to a lack of consensus regarding the role of prophylactic versus therapeutic blood transfusions. Prophylactic transfusion programs are intended for pregnant SCD patients at an elevated risk of complications, including patients with previous perinatal mortality or severe anemia. Therapeutic transfusions entail transfusing when indicated for severe disease manifestations with SCD-related complications or lower-than-baseline hemoglobin. There is no clear hemoglobin (Hb) threshold for transfusions. However, the goal is to decrease the percentage of Hb S to about 40% while increasing the total Hb level to about 10 g/dL.^9^^,^^10^

Currently, conflicting data from multiple studies has led to the recent guidelines issued by the American Society of Hematology recommending either prophylactic transfusions at regular intervals or standard care. According to these guidelines, there was insufficient evidence to favor one over the other. However, prophylactic transfusions are recommended at regular intervals at the onset of pregnancy in patients who had severe SCD-related complications before pregnancy (including in previous pregnancies) or in cases of high-risk pregnancies as in the setting of other comorbidities or nephropathy. Regular transfusions were also recommended in patients who develop SCD-related complications during an ongoing pregnancy.^11^

With contradictory evidence and conditional recommendations in current guidelines, there is a continuing debate surrounding prophylactic blood transfusions in pregnant SCD patients. The aim of this study was to retrospectively assess if scheduled red cell partial exchanges (RCPE) impacted lab values, the number of hospital and emergency department visits, as well as prenatal, perinatal, and postnatal outcomes.

## Methods

This retrospective observational cohort study was conducted at a tertiary care teaching hospital in northwestern Louisiana, USA. Upon obtaining the institutional review board's approval, we identified 47 adult pregnancies in patients with severe SCD (45 Hb SS plus 2 Hb Sβ0-thalassemia patients) spanning from April 2012 to January 2024. Patients with Hb SC and Hb Sβ+-thalassemia genotypes were excluded due to the less severe nature of the disease. Patients who received RCPE had one unit of phlebotomy followed by the transfusion of one unit of packed red cell every two weeks as per the protocol followed at our institution. The rationale for this protocol over full red cell exchange is due to patient convenience, the lack of requirement for large venous access, and the overall shortage of blood products. At our institution, for SCD patients, prophylactic red cell antigen matching for Rh (C, E or C/c, E/e) and K antigens are routinely performed. The blood products were leukoreduced. Extended RBC genotyping is not routinely performed.

The decision to perform partial exchanges was decided by previous pregnancy-associated comorbidities such as maternal or fetal adverse outcomes, phenotype/genotype of SCD, >3 sickle cell pain episode-related hospitalizations, history of recurrent acute chest syndrome or strokes, and physician discretion as this was not a randomized study. The partial exchanges were performed manually.

The data collected included demographics, comorbid conditions, laboratory results during pregnancy (Hb, white cell, platelet and reticulocyte counts, creatinine, ferritin, lactate dehydrogenase, liver enzymes, and bilirubin), number of emergency department visits, and hospital admissions for pain crises during pregnancy, as well as prenatal, perinatal, and postnatal outcomes. Patients were separated into two groups based on whether they received scheduled transfusions during pregnancy. Descriptive statistics (as means and percentages) and individual sample t-tests (for comparison of means between groups) were utilized to report the analyzed data. Chi-square and Fisher's exact tests were utilized to assess whether scheduled transfusions impacted prenatal, perinatal, or postnatal outcomes. A p-value of less than 0.05 was considered significant. A binary regression model was used to account for the influence of demographics and comorbidities.

## Results

The mean age in the study group was 25.09 ± 4.39 years. There were no significant differences in the mean age between the RCPE (24.36 years) and non-RCPE (25.47 years) groups (two-sided p-value = 0.440). All 47 patients were African Americans and had insurance coverage through Medicaid or private insurance. Of the 47 patients, 14 (29.78%) received scheduled RCPE with a 78.6% compliance rate (no missed exchanges). There is no documented evidence of alloimmunization in any of the patients. Only 2.38% (1 out of 42 patients) had documentation of venous thromboembolism.

In those who received partial exchanges and had a previous pregnancy (gravida >1), 4/8 (50%) had a prior miscarriage, and 1/8 (12.5%) had preeclampsia, while the rest (3/8; 37.5%) had no prior obstetric data available. In addition, 8/14 (57.14%) patients who received scheduled RCPE during pregnancy also received RCPEs before pregnancy for either stroke, acute chest syndrome, or recurrent pain crises as an indication.

In the RCPE group (before pregnancy), 5/14 (35.71%) patients were being treated with hydroxyurea, 1/14 (7.14%) with hydroxyurea and voxeletor, 6/14 (42.85%) not on any therapy while the remaining 2/14 (14.28%) patients had an unknown status or missing data. In the non-RCPE group (before pregnancy), 11/32 (34.37%) patients were being treated with hydroxyurea, 14/32 (43.75%) were not on any therapy, while the remaining 7/32 (21.87%) had an unknown status or missing data. Although some of these patients were previously on hydroxyurea, their disease was not well controlled, which led to the decision to treat them with scheduled RCPEs during their pregnancy. In the RCPE and non-RCPE groups, although not scheduled, 3/14 (21.42%) and 3/32 (9.37%) had received simple transfusions at some point during their pregnancy.

This study found that patients who received scheduled RCPE had fewer hospital admissions for painful crises (1.54 compared to 3.50; p-value = 0.032), and a higher gestational age in weeks at delivery (36.71 compared to 33.81; p-value = 0.007 - Table 1). The differences in means of other hematological and chemistry laboratory values were insignificant, although there was a high amount of missing laboratory data.

**Table 1:** t-tests show statistically significant differences with lower total number of hospital admissions for pain crises during pregnancy and for higher gestational age at delivery.

Variable	Partial exchange	n	Mean	SD	t-test	df	p-value	Effect size (Hedge's)
Hb nadir (g/dL)	Yes	14	7.44	1.41	1.32	38.00	0.098	0.43
	No	26	6.89	1.20				
Total number of hospital admissions during pregnancy	Yes	14	3.71	5.18	−0.43	40.00	0.336	−0.14
	No	28	4.46	5.47				
Total number of hospital admissions for pain crises	Yes	13	1.54	1.90	−1.91	38.63	0.032	−0.48
	No	28	3.50	4.67				
Gestational age at delivery in weeks	Yes	14	36.71	1.33	2.61	31.42	0.007	0.63
	No	27	33.81	5.47				

SD: Standard deviation; df: Degree of freedom; Hb: Hemoglobin.

Scheduled RCPE was associated with a lower incidence of neonatal intensive care unit admissions, with an unadjusted odds ratio of 0.071 (95% confidence interval: 0.008-0.632). However, there was no statistically significant association with other prenatal, perinatal, or postnatal outcomes such as preeclampsia, eclampsia, mode of delivery, intrauterine growth restriction (IUGR), or neonatal death. A binary logistic regression analysis, adjusted for demographics and comorbidities using the Charlson index, did not reveal any statistically significant associations (Table 2).

**Table 2:** There were lower rates of neonatal intensive care unit admissions in the unadjusted model. Logistic regression did not show any significant associations.

Variable		Partial exchange (no)$	Partial exchange (yes)$	Unadjusted p-value	Unadjusted odds radio (95% CI)	Adjusted p-value	Adjusted odds ratio (95% CI)
PCP	No	16	4	0.212	2.500 (0.648-9.651)	0.665	0.614 (0.067-5.589)
	Yes	16	10				
Preeclampsia	No	20	11	0.498	0.545 (0.124-2.409)	0.762	1.485 (0.115-19.108)
	Yes	10	3				
Eclampsia	No	28	14	1.000			
	Yes	2	0				
New VTE	No	28	13	0.333	0.317 (0.202-0.497)	1.000	
	Yes	0	1				
Mode of delivery	Cesarean	15	6	0.659	1.333 (0.372-4.785)	0.458	2.636 (0.204-34.007)
	Vaginal	15	8				
Preterm delivery	No	12	9	0.159	0.392 (0.105-1.497)	1.000	
	Yes	17	5				
Miscarriage	No	29	14	1.000			
	Yes	1	0				
IUGR	No	21	12	1.000	0.700 (0.117-4.179)		
	Yes	5	2				
NICU admission	No	11	12	0.011	0.071 (0.008-0.632)	0.071	33.742 (0.740-1537.792)
	Yes	13	1				
HDN	No	26	13	1.000		1.000	
	Yes	1	0				
Neonatal death	No	27	14	1.000		1.000	
	Yes	2	0				

PCP: Primary care provider; VTE: Venous thromboembolism; IAGR: Intrauterine growth retardation; NICU: Neonatal intensive care unit; HDN: Hemolytic disease of the newborn; 95%CI: 95% confidence interval.

$ Sum of the individuals may not equate to 33 (non-RCPE group) or 14 (RCPE group) due to missing data that was excluded in the analysis.

## Discussion

SCD is a single-gene disorder due to a mutation in the HBB gene (11p 15.4), which encodes the beta-globin chain of Hb. A single point mutation that leads to the substitution of valine instead of the normal glutamic acid at amino acid 7 results in the variant that produces Hb S.^12^ SCD is an autosomal recessive trait. The disease manifests only with homozygosity for Hb S, compound heterozygosity for Hb S and beta-thalassemia, or a different HBB variant that interacts with the Hb S. The most severe variants, such as Hb SS and Hb Sβ0-thalassemia, completely lack normal Hb. Major pathological processes that drive clinical disease include Hb S polymerization following deoxygenation, vaso-occlusion from the altered phenotype of red cells, hemolysis-mediated endothelial dysfunction, and sterile inflammation,^13^ which could impact the development of a normal placenta in pregnant SCD women.^14^

The aforementioned pathophysiologic processes could be worse during pregnancy due to the increased demand for oxygen by the developing fetus. Women with SCD have also been reported to have a blunted renin-angiotensin-aldosterone system, causing significantly lower plasma expansion compared to normal pregnancies, and this mechanism has been linked to preeclampsia, IUGR, and low birth weight.^15^ The consequences of sickling (such as hemolysis and endothelial dysfunction), combined with a background of sterile inflammation, hypoxia, and increased cellular adhesion, exacerbate cellular dehydration, shearing stress, and hyperviscosity. This, in turn, leads to more tissue hypoxia that leads to further sickling, perpetuating the vicious cycle.^14^

In certain pregnancies, transfusions with packed red blood cells or whole blood are used to relieve symptoms and reduce the risk of complications from acute events. The use of transfusions in pregnant patients with SCD is a complex and controversial topic that has been the subject of much debate. While current evidence is minimal, a meta-analysis of 12 observational studies conditionally stated that prophylactic transfusions may reduce maternal mortality, vaso-occlusive painful crises, and pulmonary complications in the mother, as well as perinatal mortality, neonatal death, and preterm birth in neonates.^16^ Sharif et al. evaluated the characteristics of SCD patients who required on-demand or emergency transfusions during pregnancy. They found that lower baseline Hb and a higher number of hospital admissions in the previous year predicted the need for transfusions during pregnancy.^17^ One trial in which patients were randomly assigned to receive either prophylactic transfusions of red blood cells, or transfusions for medical or obstetric emergencies showed a significant decrease in the incidence of painful crises of SCD.^18^ These findings were further corroborated by other studies.^19^^,^^20^ Prophylactic transfusions have also been associated with reduced maternal morbidity and mortality and improved fetal outcomes.^21^, ^22^, ^23^, ^24^, ^25^ In contrast, multiple other studies have shown no significant difference in maternal outcomes including perinatal and maternal mortality.^26^^,^^27^ The TAPS-2 trial was registered in the UK in 2019 and is currently enrolling patients to study the impact of transfusions in pregnant women with SCD.^28^

Anemia during pregnancy has previously been linked to premature birth,^29^ low birth weight,^30^ and postpartum depression.^31^ In addition to the usual iron, folate and vitamin B12 deficiencies seen during pregnancy, SCD can devastatingly exacerbate existing anemia to increase the frequency and severity of these complications. The current study shows higher baseline Hb in patients who received scheduled transfusions, which could explain the lower incidence of preterm births. Fewer emergency department visits and hospital admissions have also been recorded among patients who received scheduled RCPEs. No statistically significant outcome was found with respect to preeclampsia, eclampsia, the incidence of venous thromboembolism, mode of delivery, miscarriage, IUGR, hemolytic disease of the newborn, or neonatal death in the present study. A recent French study revealed no difference in the frequency of preeclampsia, eclampsia, HELLP (hemolysis, elevated liver enzymes, low platelet count) syndrome, term of delivery, births less than 37 weeks, birth weight, or cesarean section. Nonetheless, the results showed an independent protective effect of the transfusion program on prematurity before 34 weeks and a composite of obstetric criteria,^32^ partially in line with the results of this study.

Despite its importance, transfusion therapy has several adverse effects, including blood-borne infections, hyperviscosity syndrome, volume overload, iron overload, and alloimmunization.^33^ No documented evidence of alloimmunization was found in any of the patients in this study, including in those who received scheduled RCPEs.

This study had a small sample size of 47 pregnant patients with severe SCD, yet some results are of statistical significance. While one of the objectives was to assess the impact of transfusions on laboratory tests, a significant amount of missing data, which is expected with retrospective chart-review-based studies, was encountered. Moreover, a small proportion of patients who received scheduled RCPEs also received simple transfusions, which could have impacted the study results. Manual partial exchanges are of particular benefit in low-resource settings who are unable to perform full/automated exchanges. Hence, this modality of exchange transfusion deserves future investigation. With careful consideration and shared decision-making, scheduled RCPEs or on-demand transfusions could play a role in high-risk patients who have had maternal or neonatal complications during a previous pregnancy, and in those who have had multiple emergency department visits or hospital admissions before or during a prior pregnancy. Larger scale trials or multi-center studies, especially from areas of high SCD prevalence, are necessary to solidify evidence and to close knowledge gaps.

## Conclusions

Patients with SCD who have had complications in previous pregnancies such as preterm deliveries or high hospitalization rates could benefit from scheduled RCPE or simple transfusions. Due to the limited sample size in the twelve observational studies outlined in current guidelines, there is a need for larger, multi-center studies, especially in areas with a higher prevalence of SCD. High-quality data from further trials and multi-center studies could drive positive changes in the clinical practice and policy pertaining to transfusional support in pregnant patients with SCD.

## Ethical disclosures

This study required approval from our institutional review board (Study ID: STUDY00001880).

## Funding and financial disclosures

This study was not funded and the authors have no financial interests or relationships to disclose.

## Authors' contributions

The concept and design of this study were provided by PR. Data collection was done by AA, SJ, VG and KL. AA was involved in data management and analysis. AA, SJ and MG drafted and revised the original draft of the manuscript. Critical review was done by TB, SM, RM and PR.

## Conflicts of interest

None declared.
